# Rapid Rescue From Hyperammonemic Coma After Valproic Acid Poisoning: Dual Therapy With Continuous Renal Replacement Therapy and L-Carnitine Supplementation

**DOI:** 10.7759/cureus.15968

**Published:** 2021-06-27

**Authors:** Shyam Kiran Gandam Venkata, Kenneth Guillotte, BreeAnna Murphy, Sai Sruthi Bhuram, Sudeep Chakravarthy Bhuram

**Affiliations:** 1 Critical Care Department, Springfield Clinic, Springfield, USA; 2 Pulmonary and Critical Care Medicine, Southern Illinois University School of Medicine, Springfield, USA; 3 Emergency Medicine, Southern Illinois University School of Medicine, Springfield, USA; 4 Internal Medicine, Sri Venkata Sai Medical College, Mahabubnagar, IND; 5 Internal Medicine, Kamineni Institute of Medical Sciences, Nalgonda, IND

**Keywords:** valproic acid toxicity, ammonia, cerebral edema, hyperammonemia, coma, poisoining, sodium valproate

## Abstract

Valproic acid is a commonly prescribed drug used in various conditions including seizures, bipolar disorder, mood disorder, and migraine headaches. Confusion and lethargy among patients on valproic acid need urgent attention as it can cause increased levels of ammonia, which can lead to the development of cerebral edema and even cerebral herniation in severe cases. Here, we describe a case of hyperammonemic coma induced by valproic acid toxicity. The condition was rapidly resolved using dual therapy involving extracorporeal removal and levocarnitine.

## Introduction

Valproic acid is a carboxylic acid initially approved by the Food and Drug Administration (FDA) in 1978 [1]. It is used in its various formulations to treat complex partial seizures, absence seizures, bipolar mania, and prophylaxis of migraine headaches. It is thought to work by increasing gamma-aminobutyric acid (GABA) levels in the brain [2]. Therapeutic serum concentrations usually range between 50 and 100 µg/mL, depending on the condition being treated [3]. Life-threatening complications of valproic acid toxicity such as central nervous system depression, respiratory depression, metabolic acidosis, hyperammonemia, and liver failure can occur across a range of serum concentrations but are strongly associated with serum concentrations of >450 µg/mL [4]. Valproic acid leads to hyperammonemia by increasing serum propionic acid levels, which inhibits carbamoyl phosphate synthase, a rate-limiting enzyme in the metabolism of ammonia via the urea cycle [5]. Although the ideal treatment for valproic acid toxicity is unknown, treatment modalities previously shown by retrospective analysis to be helpful include carnitine supplementation, activated charcoal, and renal replacement therapy [6,7]. We describe the use and role of dual therapy with renal replacement therapy and levocarnitine (L-carnitine) for the rapid rescue of acute valproic acid toxicity-induced hyperammonemic coma following an intentional medication overdose.

## Case presentation

**Figure 1:**
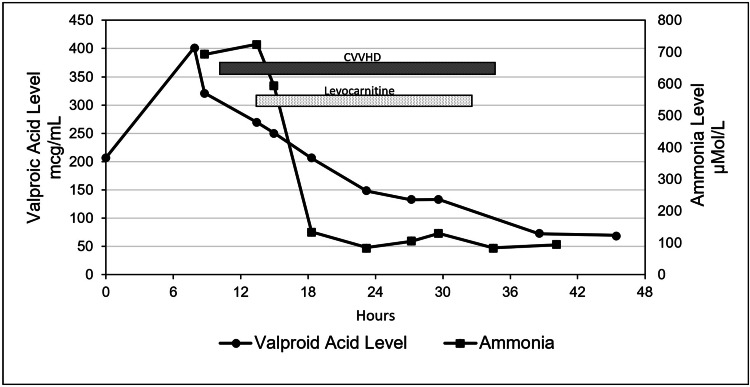
Timeline depicting valproic acid and serum ammonia levels in relation to the initiation of CVVHD and levocarnitine supplementation. CVVHD: continuous venovenous hemodialysis

A 57-year-old with a history of bipolar disorder presented to the Emergency Department (ED) after reportedly consuming two handfuls of his prescribed 500 mg delayed-release valproic acid tablets about 30 minutes prior to arrival. The patient had a brief period of alertness in the ED followed by drowsiness, combativeness, and progressive deterioration of mental status. Despite prompt treatment with gastric decontamination, the valproic acid level increased to >450 µg/ml and the ammonia level reached >700 µmol/L. The patient developed hyperammonemic coma requiring endotracheal intubation for airway protection. Considering the risk of cerebral edema due to hyperammonemia, dual therapy with L-carnitine and renal replacement therapy using continuous venovenous hemodialysis (CVVHD) was promptly initiated in the intensive care unit. The intravenous (IV) administration of L-carnitine 2,000 mg was followed by weight-based IV administration of 1,625 mg every four hours. After the initiation of L-carnitine and CVVHD, there was a rapid decline in ammonia levels and valproic acid levels (Figure 1), along with dramatic improvement in mental status. CVVHD and L-carnitine were discontinued after about 24 hours due to satisfactory improvement in mental status and laboratory profile of ammonia and valproic acid levels. He was then given lactulose and rifaximin for hyperammonemia maintenance therapy which was later tapered down and discontinued. He was extubated on hospital day three. He was then transferred to a general floor where his condition continued to improve. Psychiatry was consulted for his bipolar disorder and medication overdose. He was discharged home on hospital day nine.

## Discussion

Acute intoxication due to valproic acid most often results in mild central nervous system (CNS) depression; however, it can occasionally lead to coma and/or cerebral edema. The progression of CNS depression is usually rapid and unpredictable as the serum concentration of valproic acid does not correlate well with clinical severity. Cerebral edema secondary to valproic acid overdose can result in early herniation and ischemia. The mechanism of valproic acid toxicity causing cerebral edema does not correlate well with the dose ingested and is likely due to the accumulation of metabolite 2-en-valproate in the brain [8]. Likewise, the serum concentration of ammonia is known to correlate poorly with neurologic symptoms; however, serum ammonia levels of >150-200 µmol/L are associated with increased intracranial pressure and brain herniation [9]. Therefore, healthcare providers should not hesitate to pursue rapid rescue of patients with elevated ammonia levels in the setting of valproic acid toxicity.

L-Carnitine, an amino acid derivative, is an essential cofactor in the beta-oxidation of fatty acids. It is postulated that L-carnitine supplementation may increase the beta-oxidation of valproic acid, thereby limiting cytosolic omega-oxidation and the production of toxic metabolites that are involved in liver toxicity and ammonia accumulation [6]. In L-carnitine deficiency, products of omega-oxidation further inhibit carbamoyl phosphate synthase causing increased accumulation of serum ammonia. Replacement of carnitine, therefore, should decrease the accumulation of ammonia in the setting of valproic acid overdose [10].

In cases of life-threatening hyperammonemia, renal replacement therapy should be considered for rapidly decreasing serum ammonia concentration and improving clinical symptoms [11]. Ammonia is a small molecule that does not exist in a significantly protein-bound state, which makes it amenable to dialysis. In contrast to intermittent hemodialysis, CVVHD provides continual clearance of ammonia along with its production. CVVHD also provides the benefit of continuous control of serum sodium, pH, and fluid balance which are of utmost importance in a patient with cerebral edema [11]. Theoretically, simultaneous CVVHD and L-carnitine supplementation helps decrease ammonia levels, clear valproic acid, and concurrently curb the production of ammonia.

Several other minimally invasive treatments are used as adjunct therapies in the treatment of hyperammonemia and cerebral edema. Hypothermia may decrease some of the metabolic effects of ammonia by decreasing free radical formation, astrocyte swelling, and inflammation. Mannitol is known to be useful in reducing cerebral edema and improving mortality. Although lactulose may or may not help in the setting of acute hyperammonemia, it is a relatively low-risk treatment option. Additionally, sodium phenylacetate and sodium benzoate are thought to help eliminate serum ammonia by alternate metabolic pathways [9].

## Conclusions

We presented a case of an acute intentional overdose of valproic acid which led to life-threatening hyperammonemic coma. Successful treatment with L-carnitine supplementation and prompt initiation of CVVHD resulted in a rapid decline in serum ammonia levels. The patient was eventually discharged home and did not suffer from any known sequelae from this treatment. Although more research is needed to determine the ideal treatment regimen for acute valproic acid toxicity, early initiation of dual therapy with L-carnitine can facilitate rapid improvement.
